# Employee investigation and contact tracing program in a pediatric cancer hospital to mitigate the spread of COVID-19 among the workforce, patients, and caregivers

**DOI:** 10.3389/fpubh.2023.1304072

**Published:** 2024-01-08

**Authors:** Diego R. Hijano, Sandra R. Dennis, James M. Hoffman, Li Tang, Randall T. Hayden, St. Jude COVID-19 Case Investigation and Contact Tracing Team, Aditya H. Gaur, Hana Hakim

**Affiliations:** ^1^Departments of Infectious Diseases, St. Jude Children’s Research Hospital, Memphis, TN, United States; ^2^Department of Pediatrics, University of Tennessee Health Sciences Center, Memphis, TN, United States; ^3^Department of Human Resources, St. Jude Children’s Research Hospital, Memphis, TN, United States; ^4^Department of Biostatistics, St. Jude Children’s Research Hospital, Memphis, TN, United States; ^5^Department of Pathology, St. Jude Children’s Research Hospital, Memphis, TN, United States; ^6^Office of Quality and Patient Safety, St. Jude Children’s Research Hospital, Memphis, TN, United States; ^7^Department of Preventive Medicine, University of Tennessee Health Sciences Center, Memphis, TN, United States

**Keywords:** SARS-CoV-2, COVID-19, healthcare, healthcare personnel, occupational health, contact tracing, mitigation

## Abstract

**Background:**

Case investigations and contact tracing are essential disease control measures used by health departments. Early in the pandemic, they were seen as a key strategy to stop COVID-19 spread. The CDC urged rapid action to scale up and train a large workforce and collaborate across public and private agencies to halt COVID-19 transmission.

**Methods:**

We developed a program for case investigation and contact tracing that followed CDC and local health guidelines, compliant with the Occupational Safety and Health Administration (OSHA) regulations and tailored to the needs and resources of our institution. Program staff were trained and assessed for competency before joining the program.

**Results:**

From March 2020 to May 2021, we performed 838 COVID-19 case investigations, which led to 136 contacts. Most employees reported a known SARS-CoV-2 exposure from the community (*n* = 435) or household (*n* = 343). Only seven (5.1%) employees were determined as more likely than not to have SARS-CoV-2 infection related to workplace exposure, and when so, lapses in following the masking recommendations were identified. Between June 2021–February 2022, our program adjusted to the demand of the different waves, particularly omicron, by significantly reducing the amount of data collected. No transmission from employees to patients or caregivers was observed during this period.

**Conclusion:**

Prompt implementation of case investigation and contact tracing is possible, and it effectively reduces workplace exposures. This approach can be adapted to suit the specific needs and requirements of various healthcare settings, particularly those serving the most vulnerable patient populations.

## Introduction

Case investigation, contact tracing, isolation, and quarantine are traditional control measures used to limit the spread of infectious agents (1–5). The World Health Organization (WHO) and the Centers for Disease Control and Prevention (CDC) recommended scaling up and training a large workforce to collaborate across public and private agencies to isolate infectious cases and ensure contacts self-isolate to stop SARS-CoV-2 transmission (6, 7).

Contact tracing success depends on a well-trained workforce with sufficient resources to act quickly (8, 9). Contact tracing can be done in several ways. Forward-tracing protocols seek to identify and isolate individuals who may have been infected by the known case, preventing continued transmission through quarantine of contacts. In contrast, backward tracing backward contact tracing (BCT) is a method of contact tracing which aims to find primary or source cases and other cases that are linked to that source can be applied when a case does not know where the illness may have been acquired. It can aid in finding clusters and could reduce the size of superspreading events (10–12). Combining both strategies, hybrid or bidirectional contact tracing has been shown to have greater potential at mitigating spread of SARS-CoV-2 (13). The CDC outlined training, team components, and performance metrics to evaluate and enhance the process (7, 8). Real data and modelling have been used to assess the role of these metrics in curbing SARS-CoV-2 transmission in communities, healthcare facilities, nursing homes, and schools, and their effect on preventing hospitalizations and deaths as well as to monitor and contain cases as restrictions eased (14–24).

The role of asymptomatic infection in viral spread was recognized early in the pandemic, leading to multi-faceted that included testing of asymptomatic individuals. Expanding testing of close contacts enabled detection of a large burden of asymptomatic infection, and allowed for isolation of infected individuals at an early stage, interrupting viral transmission (25). Widespread low viral load of SARS-CoV-2 was shown by Vimercati et al. among asymptomatic hospital workers (26). As availability of testing increased, its use to decrease post-quarantine transmission and shortened the quarantine period was implemented (27). With these tools and knowledge, some countries aimed to mitigate SARS-CoV-2, often referred as “flattening the curve,” while eithers sought to eliminate the virus, an approach known as “zero COVID-19 strategy” (28–31). The focus of the latter was on eliminating the spread of the virus through the implementation of strict public health measures, followed by a phase of containment during which economic and social activities were allowed to resume while public health measures were employed to prevent any new outbreaks from spreading widely (32). Governments that decided to utilized all means possible, from closing schools and shops, to implementing strict lockdowns or even culling animals deemed to carry the virus, in order to get the cases down to zero have fared better than countries that opted for mitigation, while it effects on the economy and civic liberties has remained a topic of discussion (28, 29). However, as more contagious variants of concern, such as delta and omicron, spread quickly, the zero COVID-19 strategy, along case investigation and contact tracing in the community became difficult for public health agencies, and many countries phased-out from these. The CDC suggested jurisdictions prioritize case investigation and contact tracing based on vulnerability, congregate settings, workplaces, and healthcare facilities, including long-term care facilities and prisons (6–8).

The impact of vaccination in preventing severe disease and mitigating overall spread of SARS-CoV-2 has been well documented (33). Higher rates of vaccination have been associated with decrease community transmission, COVID-19 associated hospitalization, and deaths (34–36). In addition, vaccination of healthcare workers was shown to be critical in mitigating nosocomial spread of SARS-CoV-2 (37, 38). Currently, a new generation of monovalent vaccines targeting an XBB.1.5, a subgroup of omicron, have been deployed and recommended (39–41). However, inequities in vaccine access and low uptake remain as key challenges in mitigating SASR-CoV-2 (42–44), which continues to continue to evolve and circulate, causing waves of infection worldwide. Most of the current variants are within the sub-omicron lineage (45). Due to high population-level immunity, there is a dissociation between number of cases and hospitalizations with older adults, those with co-morbidities, and/or who are not up to date with COVID-19 vaccinations represent most individuals needing hospitalization (46).

Here, we discuss the features and effectiveness of a COVID-19 case investigation and bidirectional contact tracing program to reduce SARS-CoV-2 transmission among healthcare workers and patients in a high-risk institution.

## Methods

### Setting

St. Jude Children’s Research Hospital (St. Jude) in Memphis, TN specializes in caring for immunocompromised children at risk of severe COVID-19. St. Jude treats children from all 50 states and from around the world. About 8,600 patients are seen at St. Jude annually, most of whom are treated on a continuing outpatient basis. The hospital has 77 beds for patients requiring hospitalization during treatment. Most of our patients are treated as outpatients and stay in one of our housing facilities with rooms specifically designed and managed by us for families of children with cancer and other diseases. St. Jude currently has over 5,000 employees. During the pandemic, the government implemented lockdowns, school, restaurant and bar closures, and mask mandates. St. Jude created a COVID-19 mitigation program to protect patients and staff. It includes controlled access, ventilation, masking, distancing, symptom screening, asymptomatic testing, off-campus testing for symptomatic cases, vaccination, case investigation, and contact tracing (37). The COVID-19 program assessment described herein was deemed exempt research by St Jude’s institutional review board with a waiver of informed consent.

### COVID-19 case investigation and contact tracing team

Hospital employees were invited to volunteer part of their time ad-honorem to assist the institution by performing case investigation and contact tracing. All participants were healthcare providers (nurses, advanced practice providers, or physicians) who expressed interest and had time every week to participate. All volunteers underwent competency training for COVID-19 case investigation and contact tracing. This included understanding patient confidentiality and privacy, medical terms and principles of exposure, infection, and symptoms, as well as interpersonal, cultural sensitivity, and interviewing skills. All team members completed: (1) online training by The Association of State and Territorial Health Officials (ASTHO) and National Coalition of STD Directors (NCSD) recommended by and developed with the CDC’s input (9), and (2) interactive training with processes specific to our institution and local public health authority. A total of 20 employees participated of this program (eight physicians; one advanced practice provider, and 11 nurses, five of whom were occupational health nurses).

A Case Investigation & Contact Tracing Lead coordinated schedules provided updates to institutional leadership (e.g., successful cases contacted, referred services), monitored calls, and reviewed documentation of data obtained for quality assurance. A case investigator (usually a physician or advanced practice provider) and contact tracer (usually a nurse) called employees who had a SARS-CoV-2 test positive, explained the need for isolation, and gathered information about work-related contacts who may have been exposed. The contact tracer then notified the identified contacts of their exposure, explained the need for self-quarantine, and monitored for symptoms while providing additional resources and support services. The team managed case monitoring, follow-up, and testing. While an investigator and tracer conducted majority of the initial interviews, all members were trained to do any role if needed and over the course of pandemic, the occupational health nurses served both as case investigators and contact tracers.

### SARS-CoV-2 testing

Starting March 25, 2020, mandatory mid-turbinate nasal swab samples were collected from all asymptomatic on-campus personnel (irrespective of their role) every 4–7 days and tested for SARS-CoV-2 RNA. Frequency of testing was higher for those who had frequent contact with patients. Sample collection was done at a central, accessible spot-on campus. A drive-through SARS-CoV-2 testing station was created for employees with COVID-19 symptoms. All samples collected by St. Jude staff were tested by PCR at St. Jude laboratories. Testing was performed using one of three test systems: the NeuMoDx^™^ SARS-CoV-2 Assay (Qiagen, Hilden, Germany), the Roche Cobas6800/8800 assay (Roche Diagnostics, Risch-Rotkreuz, Switzerland), or the altona RealStar^®^ SARS-COV-2 RT-PCR assay (altona Diagnostics, Hamburg, Germany), each of which had received emergency use authorization (EUA) by the US Food and Drug Administration (US FDA). All three methods had also undergone validation by the St. Jude Clinical COVID Laboratory and been shown to perform as expected, with comparable accuracy across all systems (37). Results were reported within 2–24 h and triggered case investigation and contact tracing. Occupational Health followed up with SARS-CoV-2 positive employees weekly until they met CDC criteria to return to work. Employees who tested positive at community labs or primary care providers were asked to report it to Occupational Health for contact tracing.

### Description of the program

Employees with lab-confirmed SARS-CoV-2 were considered infectious from 2 days before symptoms (or positive SARS-CoV-2 if asymptomatic) until the end of isolation. Cases were monitored weekly until they could return to work. Employees who were within 6 feet from a case for 15 min or more cumulative within 24 h without a mask on the St. Jude campus during the infectious period were considered work-related contacts and notified immediately about potential exposure. Employees meeting the exposure definition in the community or household were classified as community and household contacts, respectively. All contacts were quarantined and monitored weekly for 14 days. Employees with a household exposure, who could not separate from the member infected with SARS-CoV-2, were monitored for longer period, as their quarantine would start when the case completed isolation. Employees on quarantine were tested 5–7 days after exposure, and/or with any new symptoms (forward tracing). All employees with SARS-CoV-2 infection were asked about known potential exposure on and off campus, as well as high-risk activities that could have led to acquiring COVID-19, to determinate the source of transmission. If exposure was unknown, investigations of cases within same working group, department, and physical location on campus were analyzed to identify a potential common source (backward tracing).

The Case Investigation & Contact Tracing Lead presented the investigation results to a panel of five physicians, four of whom were infectious diseases specialists, to decide if work-related transmission occurred. In the presence of community-based COVID-19 transmission, a workplace exposure was assumed if the case investigation suggested it was more likely than a community-based exposure. Isolation and quarantine procedures were adjusted based on the evolving CDC recommendations over the course of the pandemic (47, 48).

During the omicron wave in the US (November 2021 – February 2022), we scaled up our program without compromising employee and patient safety by: (1) recruiting more volunteers for phone triage and non-medical tasks, (2) making data collection lean by removing collection of all variables that were not critical to reporting or follow-up of the investigations, (3) using emails to report negative SARS-CoV-2 tests, (4) creating a secure live log of new SARS-CoV-2 infections, and (5) reviewing the log and extending contact tracing hours. While we make some references to observations during the omicron wave, the overall data collection during this period was reduced to what was assessed as critical to case investigation and contact tracing and is not reported in this manuscript.

### Performance evaluation of the program

Based on the CDC proposed criteria to assess a case investigation and contact tracing program’s performance, our program’s metrics included: (1) case interviewing: time to interview from diagnosis/, time to interview from notification of positive test, time from symptom onset, time to finish investigation; (2) contact notification: contacts elicited/monitored, proportion notified, time from identification to notification; (3) contact follow-up: proportion evaluated at 7 and 14 days, proportion with symptoms evaluated within 24 h, proportion who completed self-monitoring; (4) contact tracing efficacy: percentage of new COVID-19 cases among contacts during self-monitoring.

### Data capture

The Clinical Research Systems team and Occupational Health collaborated on an internal project using the web-based REDCap^®^ application. Separate forms for case investigations, contact tracing, and follow-up were created and updated based on CDC COVID-19 guidelines. The Alerts and Notifications module in REDCap^®^ was used to send email notifications to Occupational Health when forms were completed by contact tracers or criteria were met for case and contact follow-up. Microsoft Power BI (Microsoft, Redmond, WA) was used to create reports for tracking visits, follow-ups, contact tracing, cases, and incomplete forms. Reports were tailored to meet the local Shelby County Health Department reporting requirements. The sharing of individual identifiers was kept on a need-to-know basis and to meet the local health department reporting requirements.

### Statistical analysis

Demographic and clinical data were collected and presented as frequency (%) for categorical and median (range) for continuous variables. Chi-square, Fisher’s exact, Student’s t-test, or Wilcoxon rank-sum tests were used for group comparisons. A 2-sided *p* < 0.05 was considered statistically significant. All analyses were performed using SAS 9.4 (SAS Institute Inc., Cary, NC) and R 4.2.0 (R Core Team, 2020; R: A language and environment for statistical computing, R Foundation for Statistical Computing, Vienna, Austria).

## Results

### Cases with SARS-CoV-2 infection

**Figure 1:**
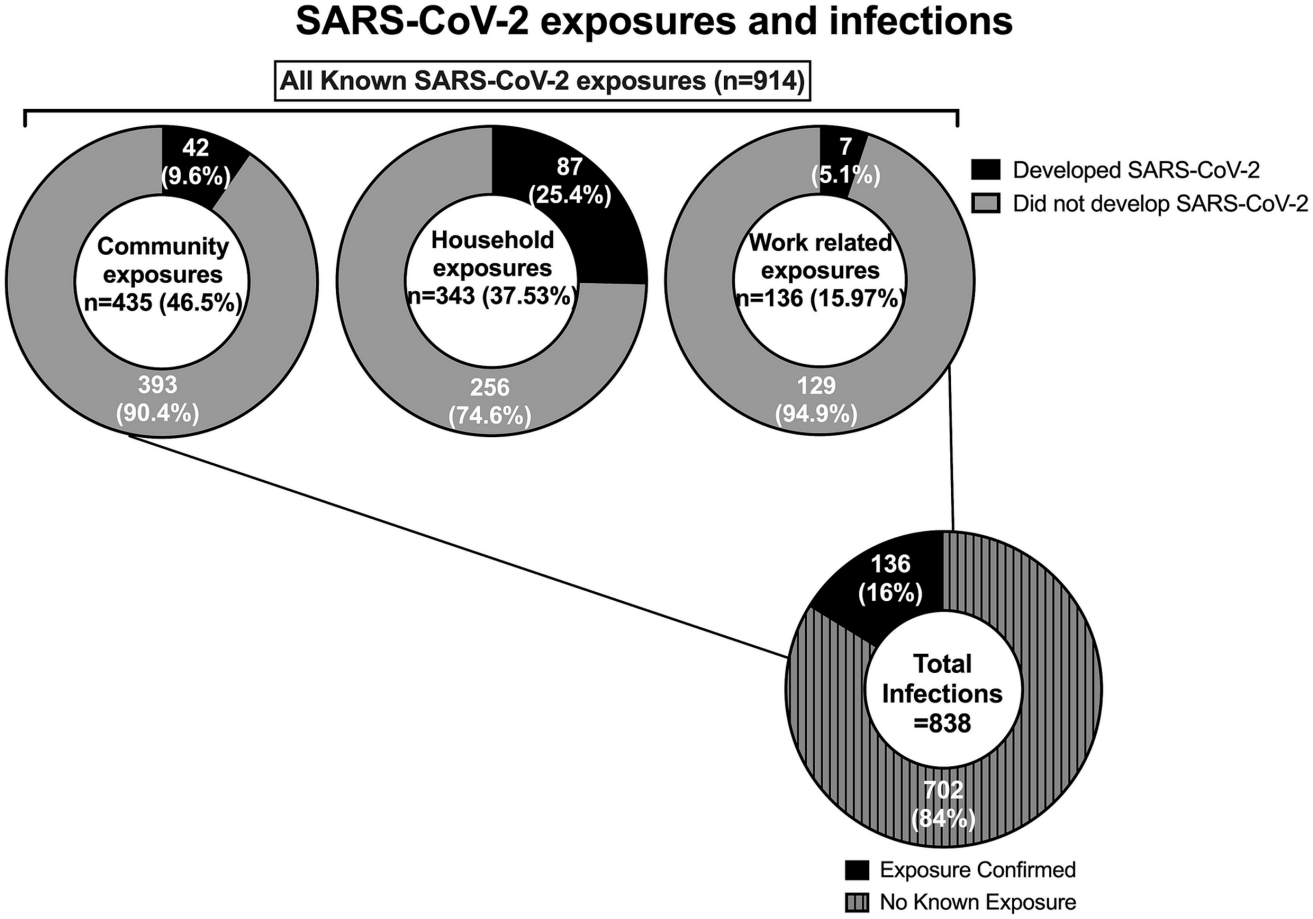
SARS-CoV-2 exposures (contacts) and infections (cases) reported to Occupational Health.

From March 19, 2020, to May 31, 2021, the program identified 914 potential exposures (778 outside the hospital campus, and 136 on campus). From these contacts, 136 employees proceeded to testing positive for SARS-CoV-2 infection during the quarantine period and were then designated as COVID-19 cases. In addition, the program identified 702 employees with SARS-CoV-2 infection for a total of 838 employees COVID-19 cases (Figure 1). Demographic information was available for 670 employees (79.95%; Table 1).

**Table 1:** Demographic information and job type of employees with SARS-CoV-2 infection.

	COVID-19 cases	
	n	%
Gender		
Female	468	69.85%
Male	202	30.15%
Age Range (years)		
18–24	28	4.18%
24–34	196	29.25%
35–44	179	26.72%
45–54	149	22.24%
55–64	107	15.97%
65+	11	1.64%
Ethnicity		
Hispanic/Latino	31	4.63%
Non-Hispanic/Latino	639	95.37%
Race		
African American	244	36.42%
Amer Indian/Alaska Native	0	0.00%
Asian	25	3.73%
Caucasian/White	360	53.73%
Native Hawaiian/Pacific Island	0	0.00%
Two or more races	10	1.49%
Other	31	4.63%
Direct Patient care		
Yes	487	72.69%
No	183	27.31%
Job category		
Advanced Practice	15	2.24%
Nursing	126	18.81%
Physician	13	1.94%
Purple	169	25.22%

**Figure 2:**
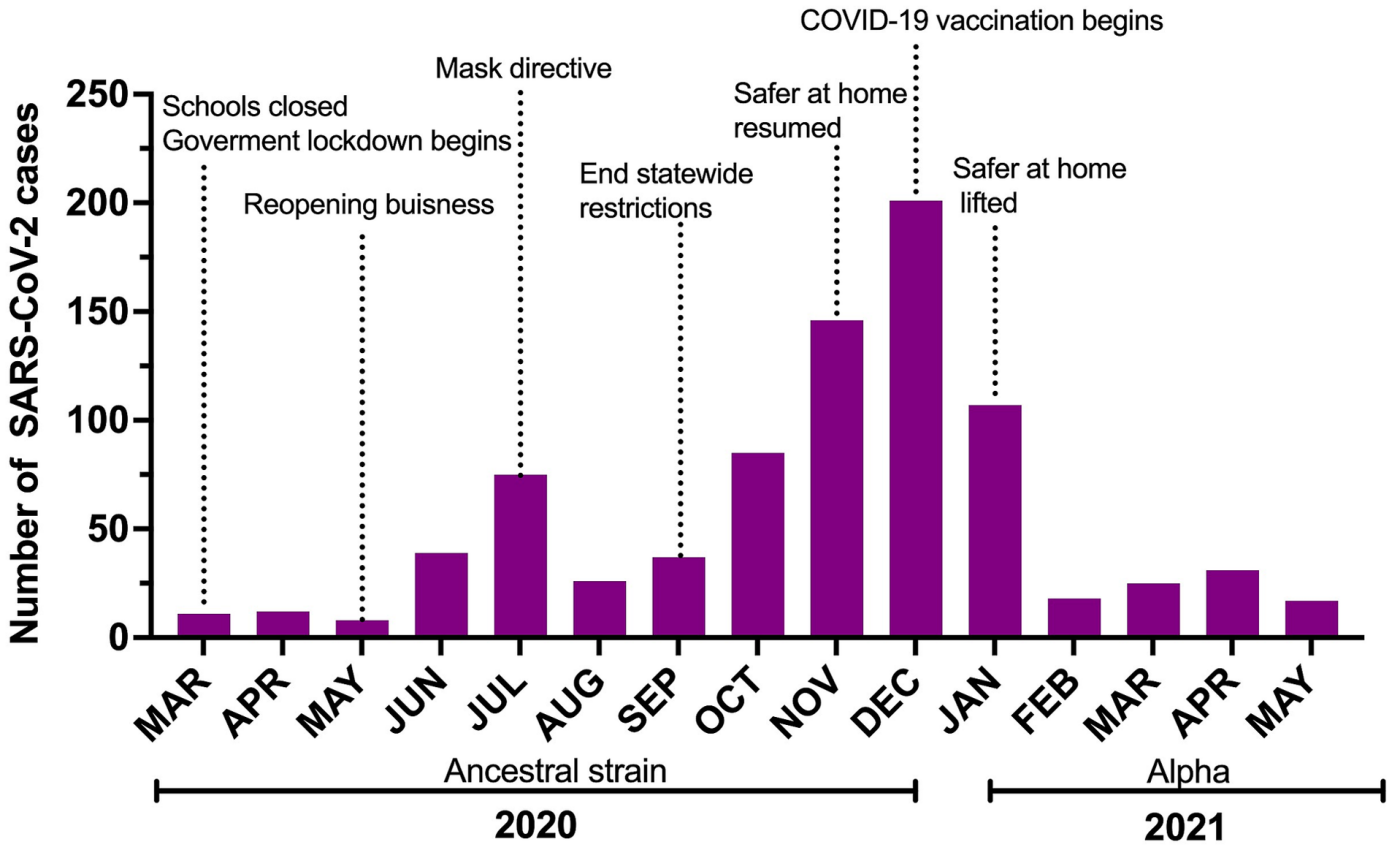
Number of SARS-CoV-2 cases reported to Occupational Health over the study period with a timeline of risk-mitigation directives and COVID-19 vaccine roll-out in Shelby County. Safer at home reflects lockdown issued by the local government.

The median number of monthly employee cases was 31 (8–201; Figure 2).

Of the 422 cases (50.42%) detected through the institutional asymptomatic routine testing program, 196 (56.44%) were symptomatic at diagnosis, 67 (15.88%) developed symptoms later, and 159 (37.68%) stayed asymptomatic until release from isolation. The median time between diagnosis and symptom onset was 1 (0–7) days for those with symptoms at interview and 3 (0–14) days for those who developed symptoms post-interview. A total of 378 (45.11%) cases were diagnosed because of the presence of symptoms and had no known exposure, including 212 (56.1%) employees at the St. Jude drive through testing station and 166 (43.9%) employees in other community testing centers. Thirty-eight employees (4.47%) were diagnosed following a known COVID-19 exposure [29 (76.32%) household contacts and nine in the community]. Seventeen of these were asymptomatic and never developed symptoms, 13 were pre-symptomatic, and eight were already symptomatic at the time of testing. Eight employees were reinfected during this period. The median number of days between episodes was 91 (26–300 days).

In this cohort, COVID-19 was mostly mild with low rates of hospitalization and complications. Only 25 employees (2.97%) developed pneumonia, and 22 (2.62%) had COVID-19 related hospitalization. Three cases (0.36%) required intensive care unit admission, and one (0.12%) mechanical ventilation. No deaths occurred during this period.

Before July 30, 2020, employees were required to have two SARS-CoV-2 negative tests before discontinuing their isolation. The median number of days from symptom onset to first negative test was 22 (14–41) days. On July 31, 2020, following CDC recommendations, the test-based approach was discontinued and replaced with a time-based approach, and the median number of days for isolation was reduced to 13 (13–15) days. These COVID-19 related isolation policies prompted a total of 12,392 days of recommended home-based isolation for our employees.

### Contacts (employees with a known SARS-CoV-2 exposure)

**Figure 3:**
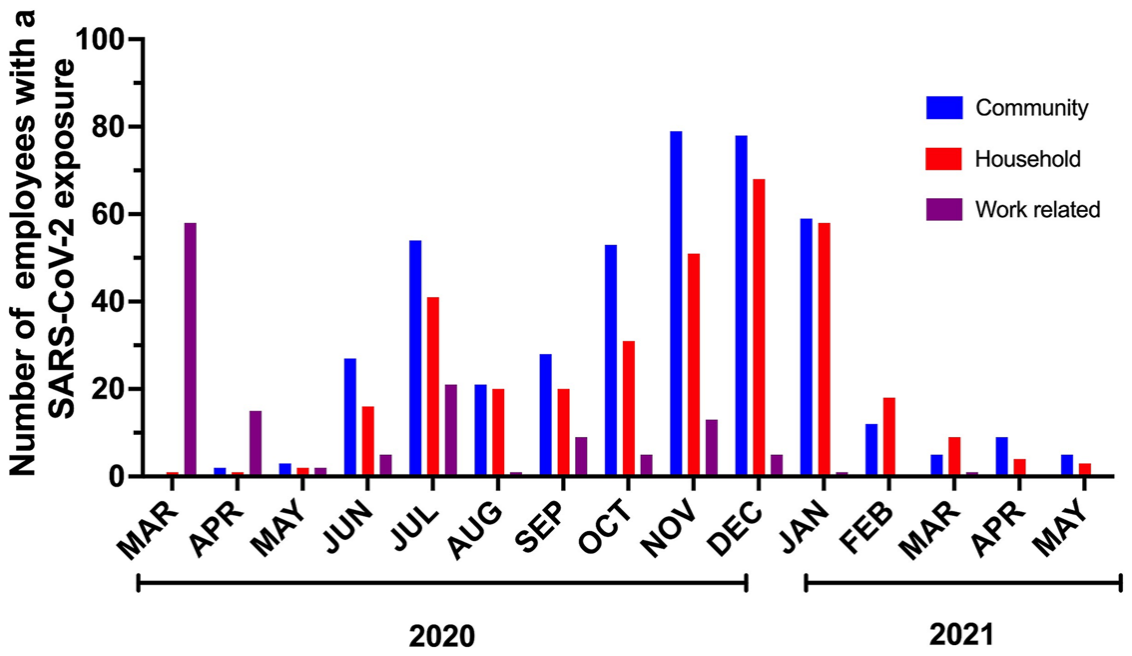
Number and type of SARS-CoV-2 exposures in employees over the study period.

914 contacts were interviewed during the study. Most (46.5%) contacts had a known COVID-19 community exposure followed by household exposure (37.53%) and work-related exposure (15.97%). We implemented universal masking 2 months after the first employee tested positive for SARS-CoV-2. This masking rule applied to everyone entering our campus irrespective of their role (employee, patient, caregiver, visitor, vendor, contractor). During this period, a shelter at home advisement for the community was established by the local public health authority, 73 out of 75 contacts were from workplace exposures, and were due to lapses in mask use and/or physical distance. As a result of routine testing, universal masking, and prompt initiation of isolation precautions when providing care to patients suspected or confirmed to have SARS-CoV-2 infections, no employee contacts resulted from exposure to patients and their caregivers. Universal masking markedly decreased the number of workplace exposures. After the initial months, most COVID-19 exposures reported by employees were from the community or home (Figure 3). Following the evolving recommendations for quarantine, the reported cohort of contacts spent a median of 14 days in quarantine following exposure to SARS-CoV-2. A total of 6,904 days were spent in home quarantine by employees with known exposure to SARS-CoV-2 who were not involved in direct patient care. In contrast, healthcare workers with direct patient care could return to work if they were asymptomatic, performed daily symptom screens, always wore masks while on campus, and underwent weekly SARS-CoV-2 testing. These employees were followed for a total of 4,439 days during the study period.

### Program performance

#### Case interviewing

More than 98% of the case investigations were initiated within 24 h of diagnosis. The median time from diagnosis to starting the interview was 0.37 h (0–24 h). When assessing the time to interview from the onset of symptoms, 58.77% reported 2 days of symptoms prior to the interview, while the rest had developed symptoms within 24 h prior to or were asymptomatic at the time of the interview. Over 97% of the interviews were completed within than 24 h from notification of diagnosis (0 days; 0–1 days).

#### Contact notifications and follow-up

All 136 workplace contacts identified during the case investigations were notified within 24 h after identification. The median time from potential SARS-CoV-2 exposure to contact notification was 2 days (0–4 days). All 914 contacts were followed during the quarantine period, with phone interviews 7 days and 14 days after exposure required for release from quarantine. 270 contacts experienced symptoms after exposure, 136 of whom were subsequently diagnosed with SARS-CoV-2 infection. Most contacts were already symptomatic at the time of notification of the exposure; 89 others (32.96%) reported symptoms during the follow-up period. Among all symptomatic contacts, 95.2% underwent SARS-CoV-2 testing, with 136 testing positive and being then designated as cases.

#### Contact tracing efficacy

A total of 136 (16.22%) employees who developed SARS-CoV-2 infection had a confirmed exposure. Seven (5.1%) employees were deemed more likely to have acquired COVID-19 in the workplace than the community, compared to 42 (30.9%) and 87 (64%) of those with community or household exposure, respectively. The seven employees who acquired SARS-CoV-2 from work-related exposure resulted from six different exposure events involving 17 employees. Exposure events involved sharing a workspace or equipment, and/or eating within six feet, with none of the employees wearing masks. All these events occurred before SARS-CoV-2 vaccines became available. No transmission from employees to patients or their caregivers, and no transmission from patients to employees occurred.

## Discussion

We detailed a successful COVID-19 case investigation and contact tracing program in a high-risk setting that reduced SARS-CoV-2 spread.

Daily monitoring of close contacts of cases can lead to faster diagnosis of suspected cases (49). In fact, rapid case detection (median time: 1 day) and contact tracing were shown to reduce virus spread (20). We noticed that tracking employees with known exposure and testing them quickly identified COVID-19 cases and enabled isolation, limiting exposure to others. Employees who tested positive for COVID-19 or had a known exposure were promptly contacted and instructed to leave the workplace immediately and not return until cleared by the company’s occupational health department..” These notifications were made within hours, so employees who posed a risk of spreading SARS-CoV-2 spent little to no time on campus which was critical to avoid staff shortages and hospital-acquired SARS-CoV-2 (50–54). We show that a program using existing resources in healthcare settings can investigate COVID-19 cases and complete contact tracing within 24 h, mostly in a few hours. This program, combined with PCR testing for asymptomatic healthcare staff, can reduce workplace transmission to staff and patients, especially in facilities that care for immunocompromised or at-risk patients. Despite the omicron wave’s rapid rise in cases, no work-related COVID-19 cases occurred, and normal operations continued due to prioritizing healthcare workers’ return to work.

Case investigation and contact tracing have been essential to reduce COVID-19 transmission, especially when testing and vaccines were unavailable (55, 56). Many groups and models have reported its effectiveness, but others have found it ineffective when used alone or when the reproductive number is greater than 2.5 (21, 24, 57). Technology-based digital apps have been used to supplement or replace contact tracing in high transmission settings (58–60). We used technology to record, report, monitor, and release COVID-19-infected and exposed employees, which allowed as to adapt and sustain our program during the Omicron wave, but never implemented a digital contact tracing app. We considered this option, but security, effectiveness, ethical, and legal issues have been raised (61). Technology can meet regulatory and medical needs, and reports can monitor pandemics and inform leaders. Whether tech-based tools help or replace traditional case investigation and contact tracing is uncertain. Thus, traditional approaches such as the one described remain important.

We reflect on a few limitations of the work we describe. Traditional contact tracing, as we describe, is subject to recall bias of cases and the case investigator’s history-gathering skills. Given the multiple institutional and local interventions that have been implemented during the pandemic, including universal masking and the COVID-19 vaccine mandate, it is difficult to isolate the impact of the case investigation and contact tracing program on its own. Therefore, its value must be evaluated in the context of existing literature that supports this approach. Strengths of the study include using observed data instead of predictive modelling to show the program’s results in high-risk settings, evaluating the program’s performance with CDC metrics, and reporting clinically meaningful outcomes. In addition, we showed that adaptability to periods of high community transmission, such as making data collection leaner, increasing the workforce, and/or using secured email for communications, is feasible to mitigate viral spread in the workplace.

With each COVID-19 wave, viral evolution, shortening of the incubation period, as well as the type of symptoms, were important challenges that led to changes in duration of isolation, quarantine, as well as recommendations about post-exposure testing (62, 63). Sumner et al. found that Omicron and Delta variants were more strongly linked to fever and cough than the original-type virus and the Alpha variant. In addition, children with an Omicron variant infection were more likely to experience lower respiratory tract symptoms and systemic manifestations (64). Similarly, Whitaker et al. noted changes in symptom patterns, with decreased reporting of loss of the sense of smell or taste for Omicron compared to previous variants, and increased reporting of cold-like and influenza-like symptoms (65). De Maria et al. reported significant differences not only in the frequency of infection among healthcare personnel, but also among the type of hospital employees who got sick, shifting from physicians early on, to nurses in subsequent waves (66). Although vaccines continue to play a significant role in mitigating SARS-CoV-2, waning immunity, along with viral evolution have prompted the need for additional doses over time, using different viral strains (34, 67). This has been particularly challenging in immunocompromised individuals who are at high-risk for severe COVID-19 and have a suboptimal response to immunizations (68, 69). Whether additional (i.e., every year) immunizations against SARS-CoV-2 with an updated vaccine formulation will become standard of care is unknown. As we continue to monitor SARS-CoV-2 dynamics, the role of case investigation and contact tracing remains to be determined.

In summary, contact tracing’s success depends on strategies, contact definitions, monitoring/reporting indicators, and data collection/analysis tools (7–9). It is resource-intensive, effective in healthcare settings and we demonstrate, feasible and sustainable. We find that while universal masking on campus had a key role in reducing at work exposure events, in addition to minimizing workplace COVID-19 exposures case investigation and contact tracing provided employee re-education/monitoring, and assurance to the workforce/patients of safeguards to minimize transmission. We share the model and performance of a case investigation and contact tracing program with a small core employee health and infection control team with the ability to rapidly expand by training eligible volunteers in a pandemic setting. Such a model can be potentially adapted for different infectious disease threats and other healthcare settings.

## Data availability statement

The raw data supporting the conclusions of this article will be made available by the authors, without undue reservation.

## Ethics statement

The studies involving humans were approved by St Jude Institutional Review Board. The studies were conducted in accordance with the local legislation and institutional requirements. The ethics committee/institutional review board waived the requirement of written informed consent for participation from the participants or the participants’ legal guardians/next of kin because this is research conducted through secondary use of data collected for occupational health purposes according to the 2018 Common Rule requirements. All analysis were done using aggregate data without identifiers.

## Author contributions

DH: Conceptualization, Data curation, Investigation, Methodology, Writing – original draft. SD: Data curation, Formal analysis, Methodology, Writing – review & editing. JH: Conceptualization, Resources, Supervision, Writing – review & editing. LT: Data curation, Formal analysis, Methodology, Writing – review & editing. RH: Resources, Supervision, Writing – review & editing. AG: Conceptualization, Methodology, Resources, Supervision, Writing – review & editing, Data curation, Investigation. HH: Methodology, Supervision, Writing – review & editing, Conceptualization, Data curation, Investigation, Resources.

## Group members of St. Jude COVID-19 case investigation and contact tracing team

Yilun Sun, Department of Biostatistics, St. Jude Children’s Research Hospital, Memphis, Tennessee, United States of America; Yin Su, Department of Biostatistics, St. Jude Children’s Research Hospital, Memphis, Tennessee, United States of America; Kari Lahmon, Department of Human Resources, St. Jude Children’s Research Hospital, Memphis, Tennessee, United States of America; Laquita Sisco, Department of Human Resources, St. Jude Children’s Research Hospital, Memphis, Tennessee, United States of America; Changhong Jiang, Department of Human Resources, St. Jude Children’s Research Hospital, Memphis, Tennessee, United States of America; Linnie Hemphill, Department of Human Resources, St. Jude Children’s Research Hospital, Memphis, Tennessee, United States of America; Jenna Gibson, Department of Human Resources, St. Jude Children’s Research Hospital, Memphis, Tennessee, United States of America; Tiffany Williams, Department of Human Resources, St. Jude Children’s Research Hospital, Memphis, Tennessee, United States of America; Gail Johnson, Department of Human Resources, St. Jude Children’s Research Hospital, Memphis, Tennessee, United States of America; Danielle Alvarado, Department of Human Resources, St. Jude Children’s Research Hospital, Memphis, Tennessee, United States of America; Linda Atwood, Department of Human Resources, St. Jude Children’s Research Hospital, Memphis, Tennessee, United States of America; Elisabeth Adderson, Department of Infectious Diseases, St. Jude Children’s Research Hospital, Memphis, Tennessee, United States of America, Department of Pediatrics, University of Tennessee Health Sciences Center, Memphis, Tennessee, United States of America; Gabriela Maron, Department of Infectious Diseases, St. Jude Children’s Research Hospital, Memphis, Tennessee, United States of America; Sheena Mukkada, Department of Global Pediatric Medicine, St. Jude Children’s Research Hospital, Memphis, Tennessee, United States of America; Liz Sniderman, Department of Global Pediatric Medicine, St. Jude Children’s Research Hospital, Memphis, Tennessee, United States of America; Craig Gilliam, Office of Quality and Patient Safety, St. Jude Children’s Research Hospital, Memphis, Tennessee, United States of America; Mary Anne Giannini, Office of Quality and Patient Safety, St. Jude Children’s Research Hospital, Memphis, Tennessee, United States of America; Tim Folse, Department of Oncology, St. Jude Children’s Research Hospital, Memphis, Tennessee, United States of America; Ali Y. Suliman, Department of Bone Marrow Transplant and Cellular Therapy, St. Jude Children’s Research Hospital, Memphis, Tennessee, United States of America; Madeline S. Wilson, Department of Hematology, St. Jude Children’s Research Hospital, Memphis, Tennessee, United States of America; Ruth Johnson, Department of Hematology, St. Jude Children’s Research Hospital, Memphis, Tennessee, United States of America; and Sri Suganda, Department of Pathology, St. Jude Children’s Research Hospital, Memphis, Tennessee, United States of America.
